# Fetuin A Concentration in the Second Trimester Amniotic Fluid of Fetuses with Trisomy 21 Appears to Be Lower: Phenotypic Considerations

**DOI:** 10.1155/2012/138971

**Published:** 2012-02-06

**Authors:** S. Iliodromiti, N. Vrachnis, Evangelia Samoli, Z. Iliodromiti, C. Pangalos, N. Drakoulis, G. Creatsas, D. Botsis

**Affiliations:** ^1^2nd Department of Obstetrics and Gynecology, University of Athens Medical School, Aretaieion Hospital, 124B Vas. Sofias Avenue, 115 26 Athens, Greece; ^2^West of Scotland Deanery, Obstetrics and Gynaecology, Glasgow, G3 8BW, UK; ^3^Department of Hygiene, Epidemiology and Medical Statistics, University of Athens Medical School, 115 27 Athens, Greece; ^4^Diagnostic Genetic Centre, 115 28 Athens, Greece; ^5^Department of Pharmaceutical Technology, School of Pharmacy, University of Athens, 157 71 Athens, Greece

## Abstract

*Objective*. We investigated whether the concentration of the glycoprotein fetuin A is altered in the second trimester amniotic fluid of trisomy 21 pregnancies compared with euploid pregnancies. *Methods*. 25 pregnancies with an extra chromosome 21 were matched for maternal and gestational age with 25 pregnancies with normal karyotype. Levels of fetuin A in amniotic fluid were measured by a commercially available enzyme-linked immunosorbent assay (ELISA) kit. *Results*. The median concentration of fetuin A in amniotic fluid of trisomy 21 pregnancies (5.3 ng/ml) was statistically significantly lower (*P*  value = 0.008) compared with that in euploid pregnancies (6.8 ng/mL). *Conclusion*. Lower levels of fetuin A in trisomy 21 may indicate an association with altered metabolic pathways in this early stage that could potentially be associated with features of the syndrome, such as growth restriction or impaired osteogenesis.

## 1. Introduction

Down's syndrome (DS) occurs in about one out of every 500 to 1000 live births [1]. The majority of the cases result from an extra copy of human chromosome 21, while the remainder are due to mosaicism or an extra part of chromosome 21. The phenotypic characteristics of DS vary significantly among individuals. Features such as short stature, reduced growth velocity [2], and reduced bone density [3] have been described in fetuses, children, and adults with DS.

Fetuin A, also known as alpha-2-HS (Heremans-Schmid) glycoprotein, is mainly expressed in the liver, the tongue and placenta in humans [4]. It occurs in high serum and amniotic fluid concentrations during fetal life [5] and is involved in development-associated regulation of calcium metabolism and osteogenesis [6]. It also accumulates in bones and teeth as a major fraction of noncollagenous bone proteins [7]. Fetuin A also plays a positive role in insulin resistance in humans and is associated with metabolic syndrome [8, 9].

We investigated whether altered concentration of fetuin A is present in the second trimester in pregnancies with trisomy 21 (DS) in order to elucidate the presence of possible metabolic pathways in utero. We hypothesized that levels of fetuin A in amniotic fluid might differ between pregnancies with DS and euploid pregnancies and that presence or absence of features in the DS group may potentially be associated with the metabolic actions of fetuin A. Fetuin A, which has been studied in maternal serum as a potential novel marker in prenatal diagnosis of trisomy 21 in the first trimester, has not been found to differ significantly in levels between euploid and aneuploid pregnancies [10]. The focus of our study is to elucidate a number of metabolic pathways in trisomy 21 rather than add new information to the already studied field of prenatal diagnosis.

## 2. Methods

The study was set up in a university hospital, a private hospital, and a genetic center. Amniotic fluid samples were collected from women who underwent amniocentesis in the second trimester of pregnancy because of advanced maternal age, pathological sonographic features, family history of inherited disorders, or abnormal first trimester biochemical screening for DS. Gestational age was estimated by an early booking scan. Multiple pregnancies and pregnancies with fetuses with gross anatomical abnormalities were excluded. Fetal karyotype was checked by QF-PCR (quantitative fluorescent polymerase chain reaction) analysis and confirmed via conventional cytogenetic cultures.

Twenty-five pregnancies having a single fetus with an extra chromosome 21 were identified and matched for maternal and gestational age with 25 uncomplicated singleton pregnancies with normal karyotype that were delivered at term. The Ethical Committee of the University Hospital approved the study protocol. Written informed consent was obtained from the participants.

Samples of amniotic fluid were centrifuged, and supernatants were stored in polypropylene tubes at −80° Celsius until the date of quantitative determination of fetuin A. Levels of total fetuin A in the amniotic fluid were measured by a commercially available enzyme-linked immunosorbent assay (ELISA) kit (BioVendor-Laboratorni medicina a.s.). The sensitivity, intra-assay and inter-assay coefficient of variation for fetuin A were 0.35 ng/mL, 3.5%, and 5.4%, respectively. Analysts were blinded to the clinical information.

For the statistical analysis, we applied the nonparametric Mann-Whitney test for the comparison of the fetuin A levels between the two groups, since the measured hormone was not normally distributed.

## 3. Results

**Figure 1:**
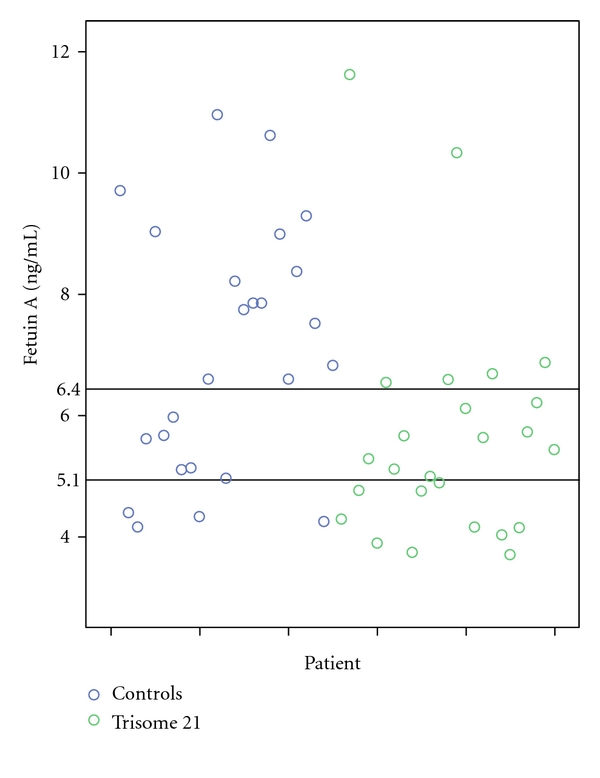
Scatter-plot graph of fetuin A concentrations (ng/mL) in normal fetuses and fetuses with trisomy 21.

Table 1 presents characteristics of the women as well as the distribution of fetuin A according to the fetuses status. Age and gestational week were similar between the two groups (*P* = 0.513, *P* = 0.216, resp.), although women that carried a fetus with trisomy 21 were slightly older and had the amniocentesis at a later stage. More specifically, 80% of the women with a DS fetus were under 40 years of age as opposed to 90% of women with euploid fetuses. The median levels of fetuin A in cases with trisomy 21 were 5.3 ng/mL as compared with 6.8 ng/mL in controls. Fetuin A was statistically significantly (*P* = 0.008 from the Mann-Whitney test) higher in amniotic fluid from euploid pregnancies compared with the levels from pregnancies with fetuses with DS.  Figure 1 presents the concentrations of fetuin A in amniotic fluid from euploid and trisomy 21 pregnancies plotted against individual patients. In addition, gestational age is correlated with fetuin A; the more advanced the gestation, the higher the concentration of the hormone in amniotic fluid for both groups (*r* = 0.44, *P* = 0.029 for euploid pregnancies and *r* = 0.55, *P* = 0.005 for DS pregnancies). On the other hand, its levels are not correlated with maternal age (*P* = 0.42). There is a considerable overlap during the early second trimester in the levels of fetuin A in amniotic fluid between euploid and trisomy 21 pregnancies, but this becomes less apparent as the gestation advances, the majority of the euploid pregnancies having a higher concentration of fetuin A regardless of gestational week.

## 4. Discussion

To our knowledge, fetuin A has not previously been studied in the human amniotic fluid. The composition of amniotic fluid is similar to that of fetal plasma up to the commencement of keratinization of the fetal skin, which starts at around 20 weeks and is completed by 25 weeks [11]. The amniotic fluid serves as an extension of the fetal extracellular compartment at least in the first half of the pregnancy. It is in direct contact with the fetal oropharynx, gastrointestinal tract, lungs, skin, and urinary system, and therefore the concentration of a fetal protein in the second trimester amniotic fluid is expected to be correlated with fetal serum concentration.

Fetuin A is a member of the cystatin “superfamily” of cysteine protease inhibitors. It is encoded by the AHSG (alpha-2-Heremans-Schmid glycoprotein) gene that is located on chromosome 3. It is mainly produced by hepatocytes and macrophages and is abundant in the bloodstream and the bones [5, 12]. Studies have shown that not only the genes located on the extra 21-chromosome of DS but also some of the genes located on the rest of the chromosomes demonstrate an altered expression compared with the euploid control group [13, 14]. Our biochemical approach revealed a statistically significant decrease in one of the final products of genes that is not directly related with chromosome 21. The exact mechanism via which the over- or underexpression of genes located on chromosome 21 can potentially affect and alter the expression of various genes on other chromosomes is still unclear; however, oxidative stress may play a role [15]. This proposal could be confirmed by measuring mRNA levels of the corresponding gene for fetuin A in amniotic fluid, and this can be a study which could form the objective of future research. In addition, since fetuin A, which is mainly a fetal protein [5], is a well-known negative acute-phase reactant whose levels decrease as a response to exogenous or endogenous stimuli [16], the reduced amount of fetuin A in the amniotic fluid derived from pregnancies with trisomy 21 may indicate important oxidative stress which can lead to various, as yet unknown, abnormal processes during this early-midfetal stage.

DS is associated with early symmetrical growth restriction, as opposed to the later onset asymmetrical growth restriction of intrauterine growth restricted (IUGR) fetuses [17]. IUGR fetuses have not been found to be associated with altered fetuin A concentrations but with defects in glycosylation of fetuin A that possibly alter its function and potentially lead to fetal growth impairment [18, 19]. In our study, since we measured the concentration of total fetuin A, our results indicate that the impaired fetal growth in trisomy 21—which is mainly symmetrical—may be associated with the reduced total fetuin A production and concentration. However, it cannot be excluded that the impaired fetal growth in trisomy 21 may also be attributed to other factors related to fetuin A, such as a defect in its metabolism or glycosylation in the early second trimester. The latter issue, which was beyond the scope of our study, remains a question to be resolved.

The action of fetuin A has been associated with bone formation and remodelling [20], and its levels correlate with the concentration of calcium during bone development [21]. Reduced levels of fetuin A in DS fetuses may have some association with the impaired bone mineralization during fetal life which could lead to delayed mineralization of the nasal bone and short femur or to the reported reduced bone density and osteoporosis in children and adults with DS [3]. Further studies are needed to elucidate this matter.

Fetuin A has been identified in early differentiating neocortical cells in numerous mammalian species [22, 23]. These animal studies so far tend to indicate that fetuin may play a significant role in neocortical development. Dziegielewska et al. have suggested, based on the drop in the levels of neocortical fetuin along with delivery, that fetuin may be responsible for early maturation of the initial layers of neocortex and the survival of cells that undergo apoptosis as part of the brain development process [22]. Although fetuin has not been examined in neocortical cells of human fetuses, postmortem examinations of DS fetuses have shown smaller neocortical cell numbers, by around one-third, in DS fetuses at 19 weeks gestation compared to the control group [24]. The lower levels of fetuin A in amniotic fluid of DS fetuses may be associated with the impaired development of the neocortex in these fetuses and with the subsequent lower mental capacity of children and adults with DS.

## 5. Conclusion

Our findings support the hypothesis that certain features of DS may be associated with altered concentrations of fetuin A in the early 2nd trimester amniotic fluid. Our data, although limited, could prompt further research into fetuin A and other proteins related to metabolism in the first half of pregnancy that should result in a better understanding of the physiopathological changes associated with DS. However, we are far from developing in utero treatments that will regulate factors that are reduced or increased with the aim of improving the pathological features of DS. Nonetheless, this approach may be of particular public health importance, especially in countries or individuals where the termination of pregnancy for DS is not legal or acceptable.

To our knowledge, this is the first study to measure the concentration of fetuin A in human amniotic fluid. Although there are limitations to our study, our data are likely to contribute to elucidation of the metabolic and inflammatory pathways in DS that are related to fetuin A from the early stage of the second trimester. Additional research is required to further explain the observed difference.

## Figures and Tables

**Table 1:** Descriptive characteristics of women (mean (standard deviation)) and levels (median (25th–75th percentile)) of fetuin A according to fetal status (trisomy 21 or euploid).

	Trisomy 21 fetuses, *n* = 25	Controls, *n* = 25
Maternal age (years)	35.9 (5.2)	34.9 (5.1)
Gestation (weeks)	19.3 (3.1)	18.4 (1.6)
Fetuin A (ng/mL)	5.3 (4.3–6.4)	6.8 (5.1–8.7)

## Abbreviations

DS: Down's syndrome
IUGR: Intrauterine growth restriction
AHSG (fetuin A): Alpha-2-HS glycoprotein or alpha-2-Heremans-Schmid glycoprotein.
